# Supplementary material for: Predicting visual function by interpreting a neuronal wiring diagram
Source: Nature. 2024 Oct 2;634(8032):113–23. doi: 10.1038/s41586-024-07953-5 (PMC11446822; doi:10.1038/s41586-024-07953-5)
Supplement: Supplementary file 6 — CRF and ERF predictions for individual TmY4 and TmY9 cells. Analogous to Supplementary Data 3, but for TmY target types. Shown are the top four monosynaptic pathways, the strongest pathway passing through each of the top ten intermediary types (ranking from Extended Data Fig. 7), and the trisynaptic pathway Tm1–TmY–Dm3–TmY (see the section entitled Prediction of spatial normalization). [file 41586_2024_7953_MOESM6_ESM.zip › DataS4/TmY4/720575940611905173.pdf]

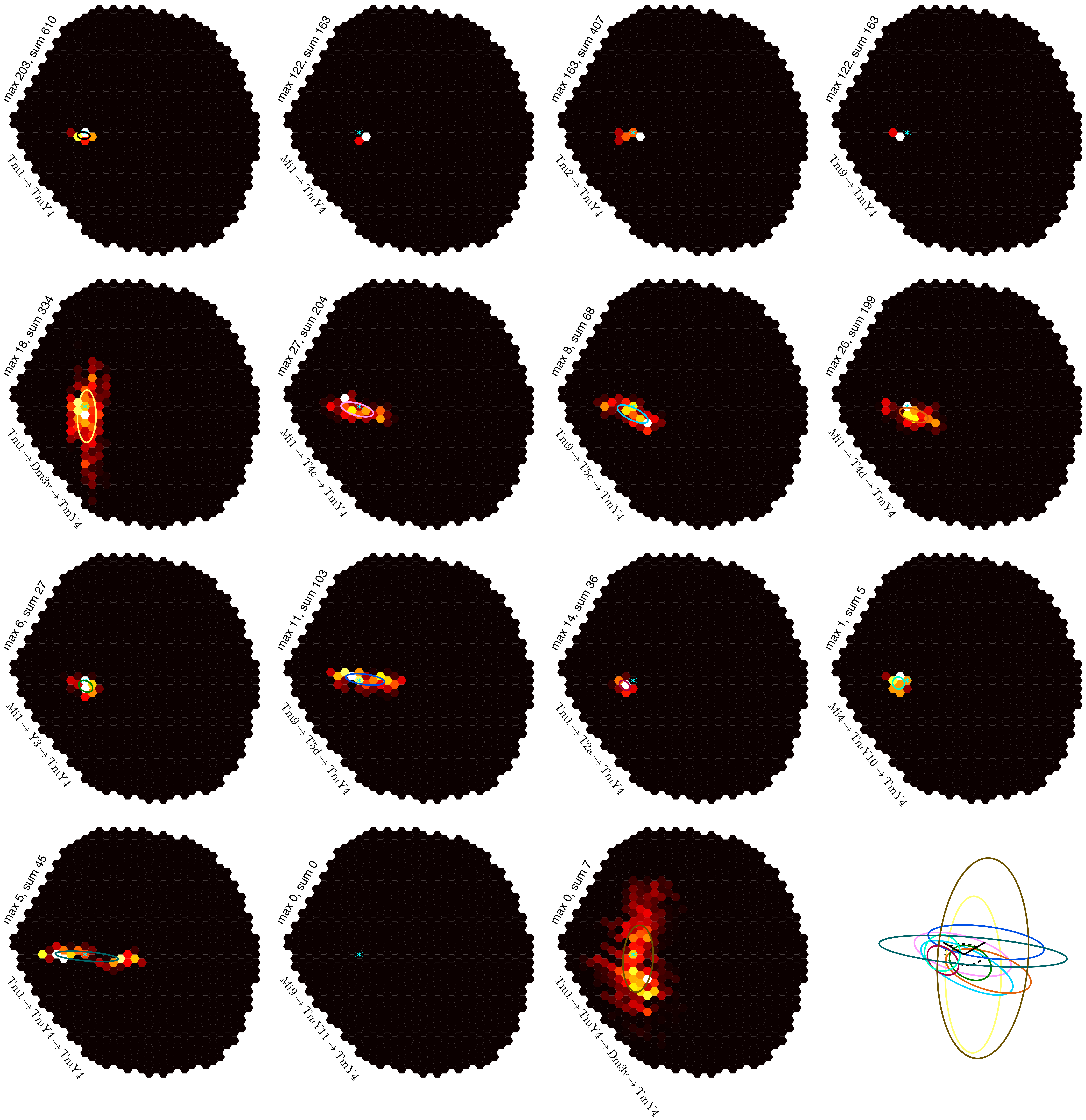
max 203, sum 610
Tm1 → TmY4
max 122, sum 163
Mi1 → TmY4
max 163, sum 407
Tm2 → TmY4
max 122, sum 163
Tm9 → TmY4
max 18, sum 334
Tm1 → Dm3v → TmY4
max 27, sum 204
Mi1 → T4c → TmY4
max 8, sum 68
Tm9 → T5c → TmY4
max 26, sum 199
Mi1 → T4d → TmY4
max 6, sum 27
Mi1 → Y3 → TmY4
max 11, sum 103
Tm9 → T5d → TmY4
max 14, sum 36
Tm1 → T2a → TmY4
max 1, sum 5
Mi4 → TmY10 → TmY4
max 5, sum 45
Tm1 → TmY4 → TmY4
max 0, sum 0
Mi9 → TmY11 → TmY4
max 0, sum 7
Tm1 → TmY4 → Dm3v → TmY4
